# How do Supervising Clinicians of a University Hospital and Associated Teaching Hospitals Rate the Relevance of the Key Competencies within the CanMEDS Roles Framework in Respect to Teaching in Clinical Clerkships?

**DOI:** 10.3205/zma000975

**Published:** 2015-08-17

**Authors:** Stefanie Jilg, Andreas Möltner, Pascal Berberat, Martin R. Fischer, Jan Breckwoldt

**Affiliations:** 1 Technische Universität München, Klinikum rechts der Isar, III. Medizinische Klinik für Hämatologie und Internistische Onkologie, München, Deutschland; 2 Kompetenzzentrum für Prüfungen in der Medizin/Baden-Württemberg, Heidelberg, Deutschland; 3 Technische Universität München, Fakultät für Medizin, MedizinDidaktisches Centrum für Ausbildungsforschung und Lehre (TUM MeDiCAL), München, Deutschland; 4 Klinikum der Universität München, Institut für Didaktik und Ausbildungsforschung in der Medizin, München, Deutschland; 5 Universität Zürich, Medizinische Fakultät, Studiendekanat, Zürich, Schweiz

**Keywords:** CanMEDS framework, competencies, clinical teaching, residency training, curriculum planning

## Abstract

**Background and aim:** In German-speaking countries, the physicians’ roles framework of the “Canadian Medical Education Directives for Specialists” (CanMEDS) is increasingly used to conceptualize postgraduate medical education. It is however unclear, whether it may also be applied to the final year of undergraduate education within clinical clerkships, called “Practical Year” (PY).

Therefore, the aim of this study was to explore how clinically active physicians at a university hospital and at associated teaching hospitals judge the relevance of the seven CanMEDS roles (and their (role-defining) key competencies) in respect to their clinical work and as learning content for PY training. Furthermore, these physicians were asked whether the key competencies were actually taught during PY training.

**Methods: **124 physicians from internal medicine and surgery rated the relevance of the 28 key competencies of the CanMEDS framework using a questionnaire. For each competency, following three aspects were rated: “relevance for your personal daily work”, “importance for teaching during PY”, and “implementation into actual PY teaching”.

**Results: **In respect to the main study objective, all questionnaires could be included into analysis. All seven CanMEDS roles were rated as relevant for personal daily work, and also as important for teaching during PY. Furthermore, all roles were stated to be taught during actual PY training.

The roles “Communicator”, “Medical Expert”, and “Collaborator” were rated as significantly more important than the other roles, for all three sub-questions. No differences were found between the two disciplines internal medicine and surgery, nor between the university hospital and associated teaching hospitals.

**Conclusion:** Participating physicians rated all key competencies of the CanMEDS model to be relevant for their personal daily work, and for teaching during PY. These findings support the suitability of the CanMEDS framework as a conceptual element of PY training.

## Zusammenfassung

**Zielsetzung: **Das Rollenmodell der „Canadian Medical Education Directives for Specialists“ (CanMEDS) findet auch im deutschen Sprachraum verbreitete Anwendung in der ärztlichen Weiterbildung. Unklar ist allerdings, inwieweit es auch auf die Ausbildung im Praktischen Jahr (PJ) in Deutschland anwendbar ist. Die vorliegende Studie untersucht daher, in welchem Ausmaß klinisch tätige Ärzte an einem Universitätsklinikum und dessen akademischen Lehrkrankenhäusern die sieben CanMEDS-Rollen bzw. deren Rollen-definierende Kompetenzen als relevant einschätzen und diese an PJ-Studierende zu vermitteln meinen.

**Methodik: **124 Ärztinnen und Ärzte aus den Fächern Innere Medizin und Chirurgie bewerteten in einem Fragebogen die Relevanz der 28 Rollen-definierenden Kompetenzen des CanMEDS-Modells („key competencies“) für folgende drei Leitfragen: „Relevanz für die persönliche tägliche Arbeit“, „Wichtigkeit für die PJ-Ausbildung“ und „Ausmaß der Umsetzung im konkreten Unterricht“.

**Ergebnisse: **Alle Fragebögen waren hinsichtlich der Hauptfragestellung vollständig auswertbar. Für die Befragten waren alle sieben CanMEDS-Rollen in der persönlichen täglichen Arbeit wichtig und wurden als relevant für die PJ-Ausbildung eingeschätzt. Für alle Rollen wurde eine Umsetzung im konkreten Unterricht angegeben. Die Rollen „Kommunikator“ (Communicator), „Sachkundiger Mediziner“ (Medical Expert) und „Teamplayer“ (Collaborator) wurden hierbei in Bezug auf alle drei Fragen statistisch signifikant als am relevantesten eingestuft. Es zeigten sich keine Unterschiede zwischen den Fächern Innere Medizin und Chirurgie, sowie zwischen Universitätsklinik und akademischen Lehrkrankenhäusern.

**Schlussfolgerung:** Die Befragten schätzten sämtliche Rollen-definierenden Kompetenzen des CanMEDS-Modells als relevant für ihre persönliche tägliche Arbeit ein. Ebenso bewerteten sie diese als relevant für die PJ-Ausbildung. Diese Befunde unterstützen die Eignung des CanMEDS-Rollenmodells als Strukturierungselement für die Ausbildung im PJ.

## 1. Introduction

### CanMEDS Roles

The framework of physicians‘ roles by the “Canadian Medical Education Directives for Specialists” (CanMEDS) [3] originally evolved from the project “Educating Future Physicians for Ontario” (EEPO) [11], which aimed at reforming physicians’ training in the sense of getting closer to conditions of real life. Data from diverse empirical sources were incorporated into this project with the result of the CanMEDS roles being anchored in all fields of the Canadian health care system [11]. The Royal College of Physicians and Surgeons of Canada transferred the framework to postgraduate medical education [5], from where it quickly spread out into many postgraduate training programmes worldwide. Examples from Germany are training programmes in surgery [7], or in general medicine [16]. A recent position paper by the “Committee for Postgraduate Training” of the German Society for Medical Education (GMA) (which introduces “entrustable professional activities”) also explicitly relates to the CanMEDS framework [2]. In some countries the model has already been applied to undergraduate education, may it be in specific teaching projects [1], [14], [18], or in national catalogues of learning objectives in Switzerland [http://www.smifk.ch, cited on March 29^th^ 2015] and the Netherlands [10]. The German “National Competency-based Catalogue of Learning objecitves in Medicine” (NKLM) also relates to the CanMEDS framework [4].

#### Background of the present study

In view of the continuum of undergraduate and postgraduate medical education the CanMEDS framework seems specifically attractive for the so-called “Practical Year” (PY) in Germany, which is located in the last year of undergraduate studies. The PY consists of three clinical clerkships of 16 weeks each and is carried out at university hospitals, or associated teaching hospitals, or other institutions with respective accreditation. Internal medicine and surgery are mandatory disciplines, whilst the third clerkship is an elective subject. With exception of the time frame PY training is highly heterogeneous. Supervision is provided by attending physicians on respective wards. The formal structure ranges from “pure attendance” to highly structured rotation plans based on logbooks, including mandatory workplace-based assessment [21]. A more homogeneous concept on a national level would therefore be desirable for PY training.

Even if the CanMEDS framework was originally developed for postgraduate training and if key competencies were only to be reached at the end of this phase, it could nonetheless be suitable for the transition from undergraduate to postgraduate training. One important precondition for this would be, that the framework (and its key competencies) was familiar to supervising physicians. With this, the continuum of medical education could be strengthened (and also the acceptance of the NKLM).

An instrument which is related to the CanMEDS model has already been used by a German research group to describe competencies of graduates from undergraduate medical education (FKM: “Freiburger Fragebogen zur Erfassung von Kompetenzen in der Medizin”) [6]. For students in the first four undergraduate years, as for postgraduate year 1 and 2 trainees, this instrument revealed good reliability and construct validity. However, it was not investigated whether clinical supervisors viewed these competencies to be relevant, and there was no explicit reference to the CanMEDS framework. 

#### Research Question

The present study was conducted to answer the following questions:

1. Do practicing physicians in one German university hospital and its associated teaching hospitals rate the 28 key competencies as relevant for:
  a) their own personal daily work?
  b) teaching during PY?
2. Do these physicians believe, that respective competencies are already taught to PY students at present?

As secondary endpoints, potential influences by the following factors were analysed: medical discipline (internal medicine vs. surgery), type of teaching institution (university hospital vs. associated teaching hospital), teacher training undergone by supervisors, number of PY students supervised so far during work life, stage of clinical training, academic position, time devoted to scientific work (by supervisors), gender, and age.

## 2. Methods

### Questionnaire

In order to reach a sufficient number of clinical supervisors, we decided to use a closed-question questionnaire instead of applying a more open, qualitative design. 

**Figure 1:**
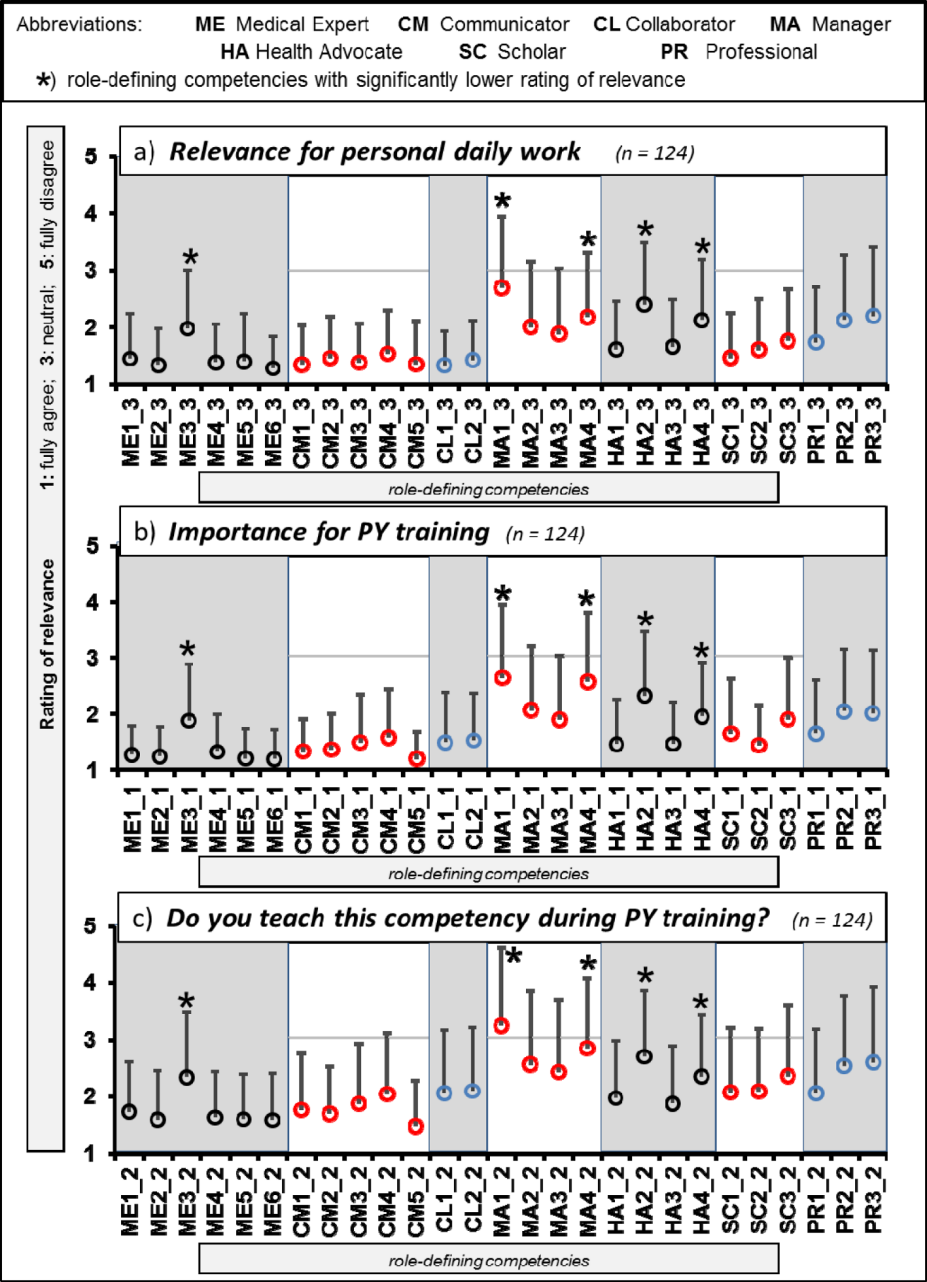
Rating of key competencies in respect to (a) relevance for personal daily work, (b) importance for “Practical Year” (PY) training, and (c) whether competencies are actually taught during PY. Depicted are means (circles) and standard deviations (bars, one direction only). The rating scale for relevance ranged from 1 (“fully agree”) to 5 (“fully disagree”).

We limited the investigation to the two mandatory disciplines internal medicine and surgery, firstly to represent the main PY subjects, and secondly to be able to look at potential differences which might be present between divergent cultures between these disciplines. The questionnaire was specifically developed for the study and included the 28 key competencies, grouped according to the respective CanMEDS roles (2 to 6 competencies per role (see table 1 (Tab. 1)); abbreviations of role names are also used in figure 1 (Fig. 1)). Descriptions of the seven roles and of the key competencies were translated to German. For role titles, we also indicated the original English expression, because we felt that translations of subject-heading-like role titles had in part missed the meaning. Official translations of the NKLM could not be used at the time of the study, since they had not been published then. At present, they are still only mentioned by an in-official report of the German “Board of Medical Faculties” (“Medizinischer Fakultätentag”) [http://www.mft-online.de/lehre/nationaler-kompetenzbasierter-lernzielkatalog-medizin, cited on March 30^th^, 2015]. For this study we used the following translations: “Kommunikator” (for “Communicator”), “Teamplayer” (for “Collaborator”), “Führungskraft” (for “Manager”), “Fürsprecher der Gesundheit” (for “Health Advocat”), “Lernender Dozent” (for “Scholar”), “Professionell arbeitender Arzt” (for “Professional”) und “Sachkundiger Mediziner” (for “Medical Expert”). The roles were characterised by the specific key competencies (see table 1 (Tab. 1)) [3], [http://www.royalcollege.ca/portal/page/portal/rc/canmeds/framework, cited on March 30^th^, 2015]. 

In our eyes, the combination of the role description with the corresponding key competencies sufficiently defined the roles. Further translation of key competencies was undertaken (by SJ) as a nearly literal interpretation of the original [http://www.royalcollege.ca/portal/page/portal/rc/canmeds/framework, cited on March 30^th^, 20154]. After retranslation into English by a person who was not involved in the primary translation (JB) ambiguities could be solved by discussion. 

Participating physicians rated the relevance of all key competencies by 5-point-Likert scales (ranging from “1”: “full agreement” to “5”: “full disagreement”). Ratings were given for the following three questions:

1. “Is this competence of relevance for your personal daily work?”
2. “Do you believe this competence to be important to be taught to PY students?”
3. “Do you teach this competence to PY students?”.

Potential confounders (medical discipline, type of institution, teacher training of supervisors, number of PY students supervised, stage of clinical training and academic position of supervisors, time devoted to scientific work, gender, age) were assessed by selection answers. At the end of the questionnaire participants prioritized the six “peripheral” CanMEDS roles (excluding “Medical Expert”) in respect to their importance for PY training. In total, the questionnaire included 90 single variables [see Attachment : supplemental material online].

#### Procedure

The survey was conducted between August and October 2012. Participating physicians were employed at the university hospital of Munich Technical University (TU München, “Klinikum rechts der Isar”), or at five associated teaching hospitals (“Deutsches Herzzentrum München”, “Klinikum Bogenhausen”, “Klinikum Schwabing”, “Krankenhaus der Barmherzigen Brüder München”, “Rotkreuz-Klinikum München”). 

From each hospital all surgical and all internal departments were included. Since some years, all institutions used logbooks to structure PY training. Further teaching was delivered by clinical seminars, mostly by experienced senior clinicians. The majority of teaching however, was given by attending physicians of different levels of experience. 

Study participation was voluntary, answering forms were anonymized. Answering sheets were filled out during the course of official staff rounds. Approximately 50% of physicians within a department attended the meetings, all attendees participated. Every department was visited only once. After a brief introduction to the study purpose and the design of the questionnaire, answering forms were filled out and subsequently collected. There was no time limit, in maximum 30 min were needed for completion. SJ was present throughout the procedure to be able to solve open questions.

#### Data management and data safety

Answers were marked on a machine readable form, and were transferred to an spreadsheet data file. For the overall rating of each CanMEDS role, we calculated the mean of the respective key competencies, results were given as means (M) with standard deviations (SD). In order to test for potential influences of confounders (medical discipline, type of institution, teacher training, number of PY students supervised, stage of clinical training and academic position of supervisors, time devoted to scientific work, gender, age) we performed analyses of co-variance with ratings of relevance as dependent variable, and the nine potential cofounders as independent variables. To ensure the alpha-level at multiple testing, an Omnibus-F-test was used. Calculation was performed by SAS, Version 9.3.

No data which could have identified an individual person were retrieved. The ethical committee of TU Munich (IRB) approved the study protocol (project no 5517/12).

## 3. Results

### Characteristics of participants

In respect to the primary study question, all 124 questionnaires could be analysed (100% response due to peer-group conduct). 60.5% of the physicians stated to have supervised between ten and 100 PY students until then, another 23.4% even more than 100. 31.5% of the physicians worked in surgery, 66.9% in internal medicine (two persons did not specify their discipline); 44.4% were female. 54.8% were employed at the university hospital, whilst 45.2% worked at one of the associated teaching hospitals. 16.1% held an academic degree similar to a PhD (called “Habilitation”), or were in the final process to obtain this degree. 6.5% had undergone relevant training in medical education (teacher training) with 120 hours, or more. Further characteristics of participants are shown in table 2 (Tab. 2). As an additional finding during the introduction session for participants it became clear, that the CanMEDS model was not explicitly familiar to most of the physicians.

#### Rating of key competencies

Single ratings of all key competencies are shown in figure 1, grouped according to:

1. “relevance for personal daily work”,
2. “importance for PY training” and
3. “teaching of the competence to PY students”.

**Figure 2:**
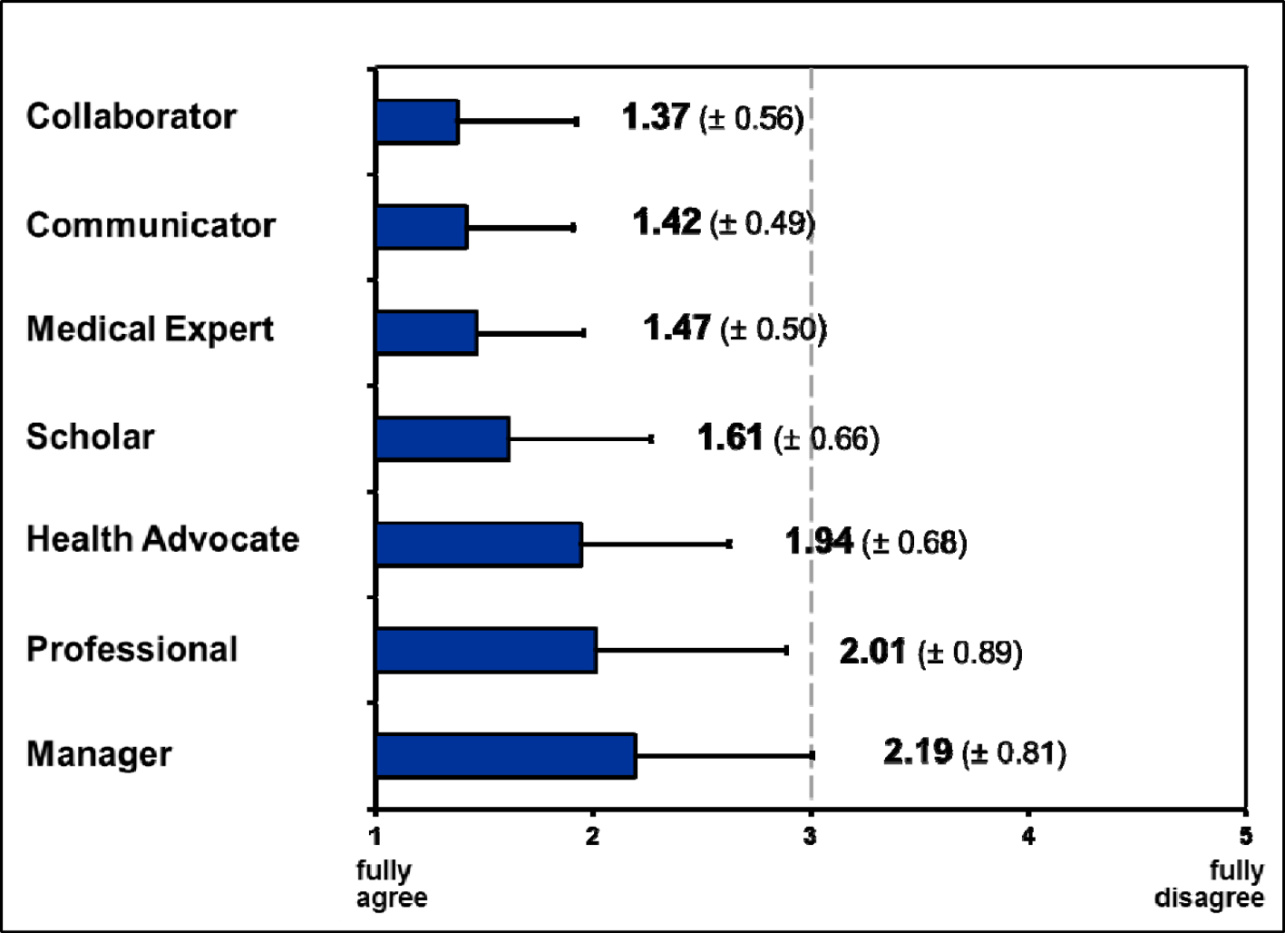
Ranking of CanMEDS roles: Relevance of roles for personal daily work, given as means from the key competencies. Depicted are mean values (boxes) and standard deviations (bars, one direction only). The rating scale for relevance ranged from 1 („fully agree“) to 5 („fully disagree“).

**“Relevance for personal daily work”** was rated higher than “neutral” for all competencies, the mean from all key competencies was 1.71±0.38. The mean of the seven CanMEDS roles ranged from 1.37±0.56 (“Collaborator”) to 2.19±0.81 (“Manager”). If compared to all other roles by paired t-tests, the relevance of the roles “Collaborator”, “Communicator” (1.42±0.49) and “Medical Expert” (1.47±0.50) was rated significantly higher (details see figure 2 (Fig. 2)).

For the question** “Importance of the compentency for PY training”** similar results were found (see figure 1 (Fig. 1)) as in respect to the relevance for personal daily work. The mean from all specific competencies was 1.67±0.41 (highest rating: 1.19±0.54, lowest: 2.65±1.31), and none of the single competencies was rated below “neutral”. For the mean values of single roles, the same roles were rated significantly more relevant as in the first question (relevance for daily work): “Medical Expert” (1.34±0.41), “Communicator” (1.39±0.47) and “Collaborator” (1.50±0.79). The role “Manager” however, was rated to be significantly less relevant (2.29±0.90).

**Figure 3:**
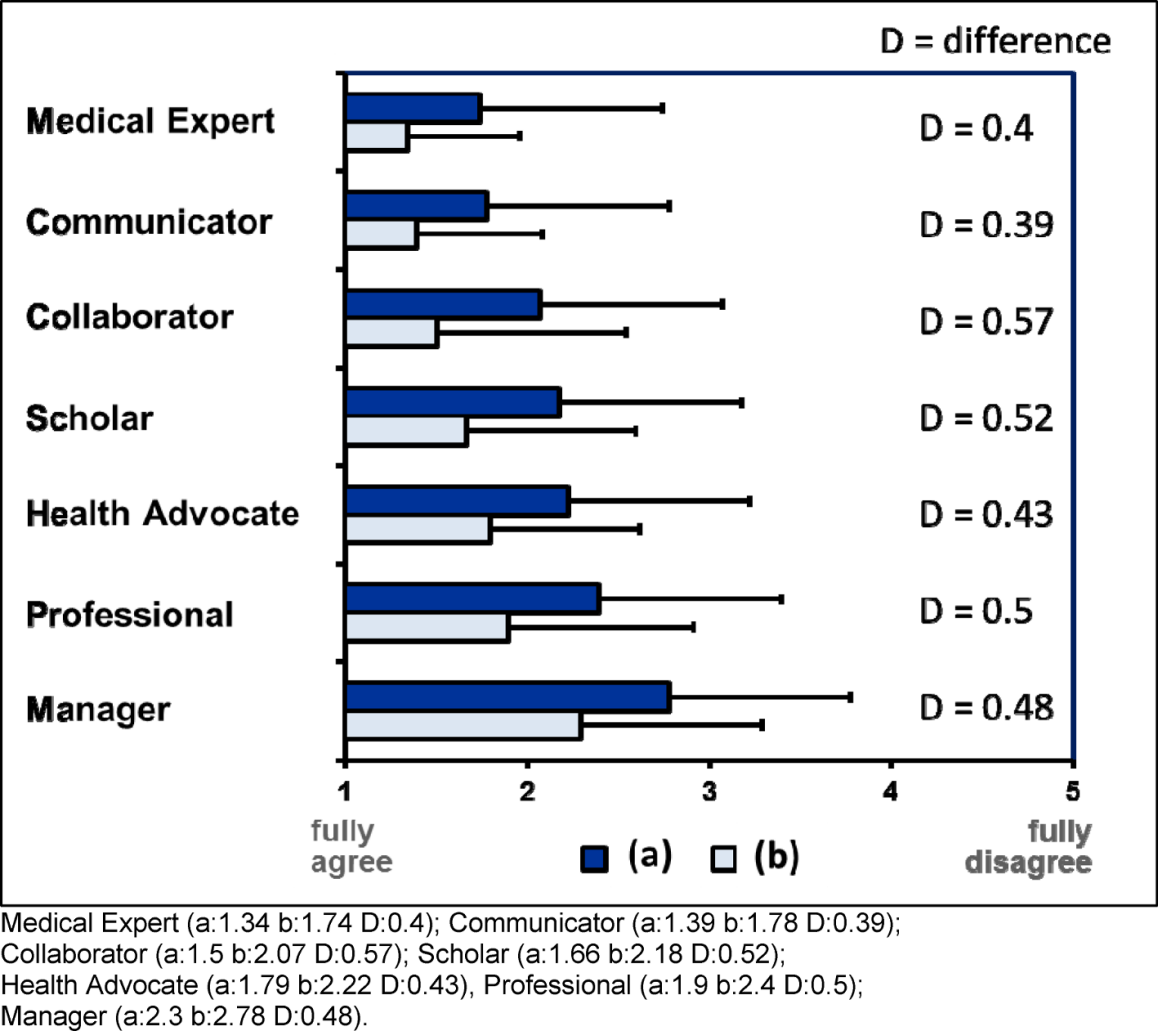
Differences (D) between (a) “importance for training during the “practical year” (PY)”, and (b) “actual teaching during PY”.

For the third question: **“Teaching of the competency during actual PY training”** again, a similar distribution was found as for the first two questions (see figure 1). Here, we found a clear difference between the perceived importance for PY teaching and the definite transfer into actual teaching (see figure 3 (Fig. 3)). The largest difference was noted for “Collaborator” (+0.57), and the lowest for “communicator” (+0.39). There were no significant differences between the seven CanMEDS roles.

Five single key competencies were rated to be significantly less relevant than all others (marked with an asterix in figure 1 (Fig. 1)). These were “Seek appropriate consultation from other health professionals, recognizing the limits of their expertise” (role: “Medical Expert”), “Participate in activities that contribute to the effectiveness of their healthcare organizations and systems” as well as “Serve in administration and leadership roles, as appropriate“ (both in „Manager”), “Promote the health of individual patients, communities, and populations” and “Respond to individual patient health needs and issues as part of patient care” (“Health Advocate”).

**Figure 4:**
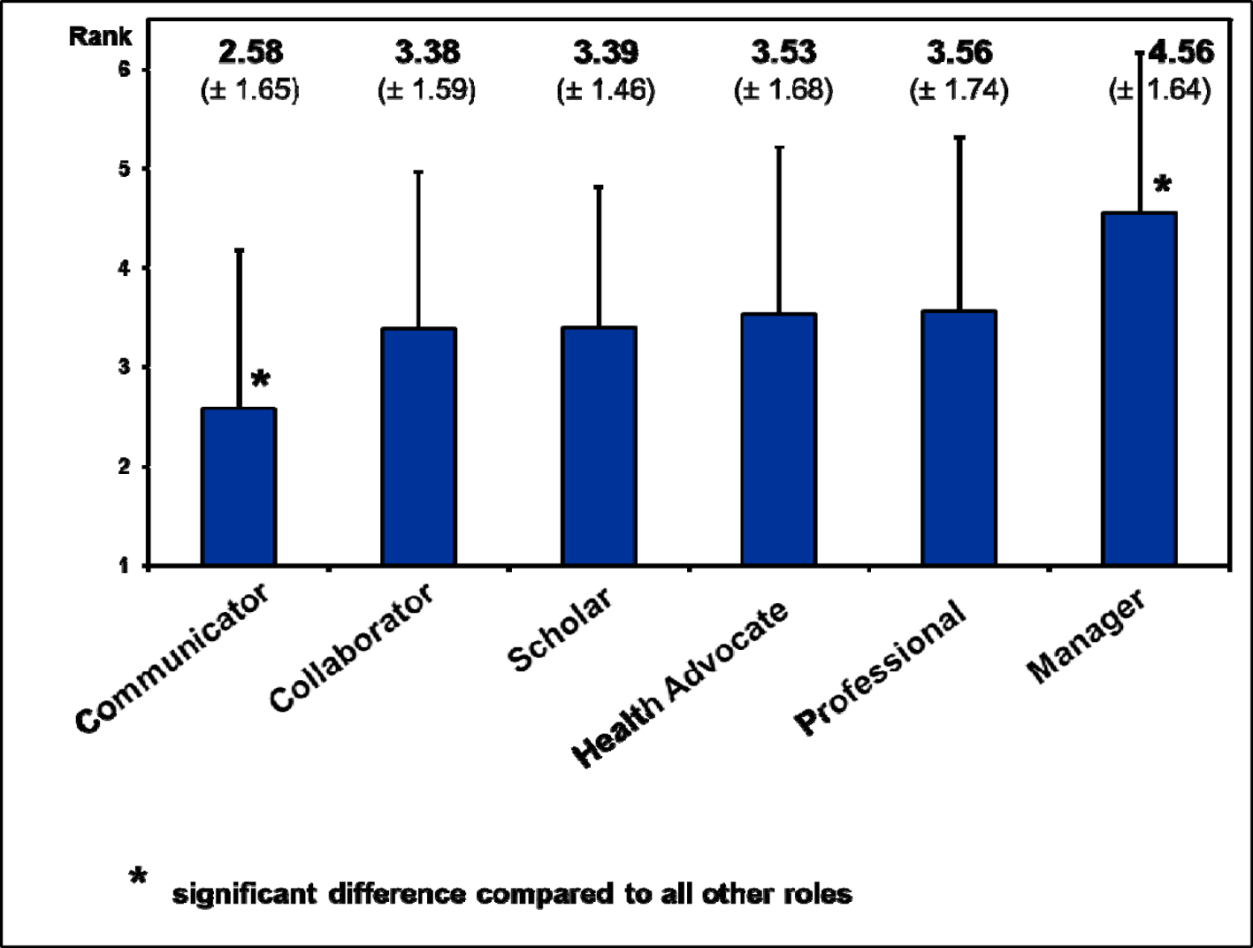
Ranking of 6 CanMEDS roles (excluding “Medical Expert”). Depicted are mean values (boxes) and standard deviations (bars, one direction only). The rating scale for relevance ranged from 1 (“fully agree”) to 5 (“fully disagree”).

In the final **ranking of roles in respect to their importance for PY training** (excluding the “Medical Expert”) the “Communicator” role was rated significantly more important than all other roles, whilst the “Manager” role was significantly less important than all other roles (compared by t-tests). We found no other statistical differences (see figure 4 (Fig. 4)).

#### Potential influences 

For none of the potential confounders (medical discipline, type of institution, teacher training, number of PY students supervised, stage of clinical training and academic position of supervisors, time devoted to scientific work, gender, age) we could show a statistically significant influence (analysis of co-variance; global test of the model: Omnibus-F-test).

## 4. Discussion

In this study 124 supervising physicians of PY students rated all seven CanMEDS roles and the respective key competencies to be relevant for their own personal daily work and for PY training. This is remarkable, as the framework was not explicitly familiar to most of the participants. Only five key competencies were rated to be significantly less relevant than all others, however all ratings were better than “neutral”. In principle, our results confirm studies from other European countries in the field of postgraduate training [15], [19]. However, these studies showed differences between hospital-based and community-based disciplines [15], and therefore it was called for additional discipline-related elements [19]. These differences could be due to higher culture dependent differentiation during postgraduate training if compared to PY training, which may have a more generic character. When asking trainees during the first two years of postgraduate training in Germany, Giesler et al. (s. introduction [6]) found very similar results as in our present study. In contrast to Ringsted [15] and van der Lee [19], Giesler et al. explicitly referred to competencies at the end of undergraduate studies.

It seems noteworthy, that physicians supervising PY students rate the roles “Collaborator” und “Communicator” as equally relevant as the “Medical Expert”. In part, comparable findings have been made in Denmark, where the “Communicator” role was rated as most important at all stages of postgraduate training [15]. For the first two postgraduate years in Germany communicational competency, team-competency, medical competency, and learning competency were rated to be the most important [6]. This emphasises the importance of non-discipline-related competencies at the workplace and calls for more attention to be paid to this field in undergraduate education [9].

Also of note is the fact, that the “Scholar” role was rated only slightly less important; this phenomenon was also reported by Ringsted [15] and Giesler [6]. Apparently, physicians perceive continuous medical education and evidence based clinical decisions as central aspects of their daily work. In our study it may have also been of relevance, that more than half of the participants was engaged in scientific work. 

The role of the “Manager” was consistently rated least important. One reason for that could be, that a minority of participants held leading positions and most participants focussed themselves on the patient-physician relationship. This lower relevance was also found by Giesler et al. [6], which throws a light on the poor representation of leadership training in German medical education [8]. In contrast, the Danish study found a higher importance of the “Manager” role. Differences in working culture could be one reason, however, the authors also modified the description of the “Manager” role in their survey by relating it more closely to the Danish health care system [15].

As already said, some of the key competencies were rated to be less important (related to the roles “Medical Expert”, “Manager”, and “Health Advocate”). These competencies address the topics interprofessional communication, basic societal conditions, leadership skills, and preventive medicine. These fields are less central to the individual patient-physician-relationship and therefore might be perceived as less important in the eyes of clinically active physicians. As an interesting point the explicitly interprofessional key competencies (under “Collaborator”) were rated to be more relevant than the key competency “ME3” (Seek appropriate consultation from other health professionals, …) under “Medical Expert”. An explanation may be that the competency “ME3” was rated in comparison to the other key competencies of the “Medical Expert”, while competencies under the “Collaborator” role were clearly limited to collaboration. 

For the lower rating of competencies related to prevention, the hospital-based background of participants could be a good explanation. This result is in line with the work of Ringsted et al [15].

**Actual teaching of competencies in PY training** was judged to be less than the importance assigned to these competencies before. This difference is similar for all CanMEDS roles. Possibly this reflects the natural difficulties to integrate teaching into the daily routine work while treating patients. In addition, some of the competencies are difficult to teach, as e.g. stated by program directors in respect to the role of the “Professional” [20]. In our study, the scales were not primarily designed to relate to each other, and also the questionnaire did not assess the teaching activities performed in reality. Therefore, no conclusion can be drawn for a possible discrepancy between “delusion” and “reality”. As an additional limitation the students’ perspective was not assessed. This would have given an insight in the competencies students had perceived to be taught. 

### Potential influences

**Medical discipline:** We did not find statistical differences of physicians’ ratings between surgery and internal medicine. Other studies demonstrated such differences if workplace contexts were clearly different, as between community-based and hospital-based disciplines [15]. However, if “technical” and “cognitive” disciplines were compared, respective differences could not be shown [15]. Another research group could not show significant differences for the appraisal of CanMEDS roles between general practitioners and specialists [17]. Therefore, it is not very likely to find discipline related differences for PY training in a hospital setting. Preconceptions in respect to differing values towards teaching between surgery and internal medicine do not hold for our sample. 

For **the type of hospital** one could have expected, that the CanMEDS framework was less established in associated teaching hospitals, and that key competencies were judged to be less relevant (based on the assumption of greater proximity to undergraduate medical education in university hospitals). This hypothesis could not be confirmed by our data. However, it cannot be ruled out, that a common working (and teaching) culture between teaching hospitals and the “home” university might have decreased such an effect. 

**Teacher training** of a volume of 120 h, or more (which may have transported the basic principles of the CanMEDS framework), did not lead to differences in the rating of single key competencies. In our view this supports the value of the concept in a sense, that it seems well anchored in daily clinical practice and does not need much additional instruction for use. At the same time, this is a retroactive confirmation of the original CanMEDS framework. 

#### Generalisability

Supervision of PY students in Germany is heterogeneous. As an example, the logbooks used in the studied hospitals are not national standard. Also, the proportion of university staff is relatively high in our study. From that perspective, our results may not be extended to a national level. On the other hand, our participating physicians represent a fairly typical cross-section in respect to clinical experience and the proportion of females (which is almost identical to recent figures in Germany: 44,4% vs. 45,0%) [13]. It is also realistic, that most physicians had not undergone teacher training. We can also exclude a selection bias due to financial motivation, since no reimbursement was given. A valuable extension of the study would have been to involve additional university hospitals and respective teaching hospitals or to community-based or rural settings. In these cases we would expect gradually different patterns with more emphasis on the “Health Advocate” role, however, a general rejection of the framework is unlikely [15]. Finally, a study of the elective discipline clerkship could extend the scope.

#### Limitations

The CanMEDS framework was not familiar to most of the participants prior to the study, so in consequence we obtained an unprejudiced, naïve picture. This is of advantage, if an intuitive understanding of the framework is tested. As a potential limitation one could argue, that a concept of “competencies” (and of competency-based education) was unknown to most participants. Even the German licensing regulations for physicians (“Ärztliche Approbationsordnung“) do not mention competencies [12]. On the other hand it is questionable, whether a deeper understanding of the concept would have led to substantially different results. At the end of the day, it would not be conducive to a wider application of the framework, if extensive training was necessary before use. 

As final and important limitation it has to be stated, that this study does not provide insight into the perspective of PY students. It is likely, that supervisors believe to teach certain competencies, whereas involved students do not perceive this in the intended way. 

## 5. Summary and conclusions

For a non-representative sample of 124 clinically active physicians we could show, that all CanMEDS roles and the respective key competencies were rated to be relevant for personal daily work, and for PY training. Results were independent from the type of teaching institution and from the background medical discipline (surgery or internal medicine). 

These results confirm, that the CanMEDS framework was well founded in the cohort of participating physicians. In view of the continuum of undergraduate and postgraduate medical education the framework could therefore serve as a structure to facilitate transition. Effects of its use should be assessed systematically, especially after implementation of the German national catalogue of learning objectives (NKLM).

## 6. Acknowledgement

We would especially like to thank all participating physicians, who devoted their time to answer the questionnaire. We also thank Mareike McIntyre for her support when planning the study. This study was conducted as a master’s thesis within the German “Master of Medical Education” (MME-D) programme. Financial support was provided by the Dean of the Faculty of Medicine, Technical University Munich and by the Department of Hematology and Oncology (III. Medizinische Klinik) of the University Hospital “Klinikums rechts der Isar”, Munich.

## 7. Competing interests

The authors declare that they have no competing interests.

## Supplementary Material

Supplemental material

## Figures and Tables

**Table 1:**
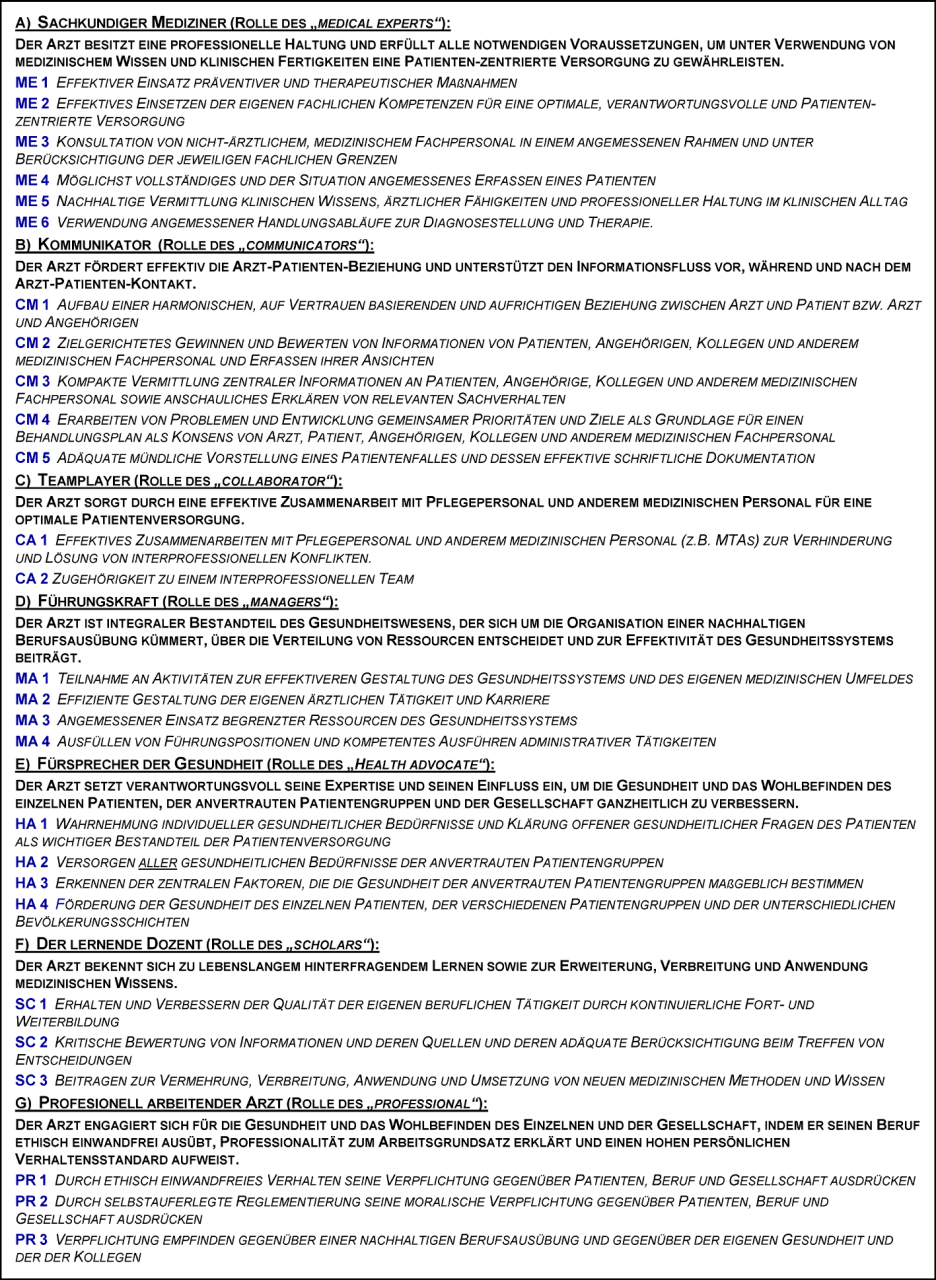
CanMEDS roles and key competencies (German translation)

**Table 2:**
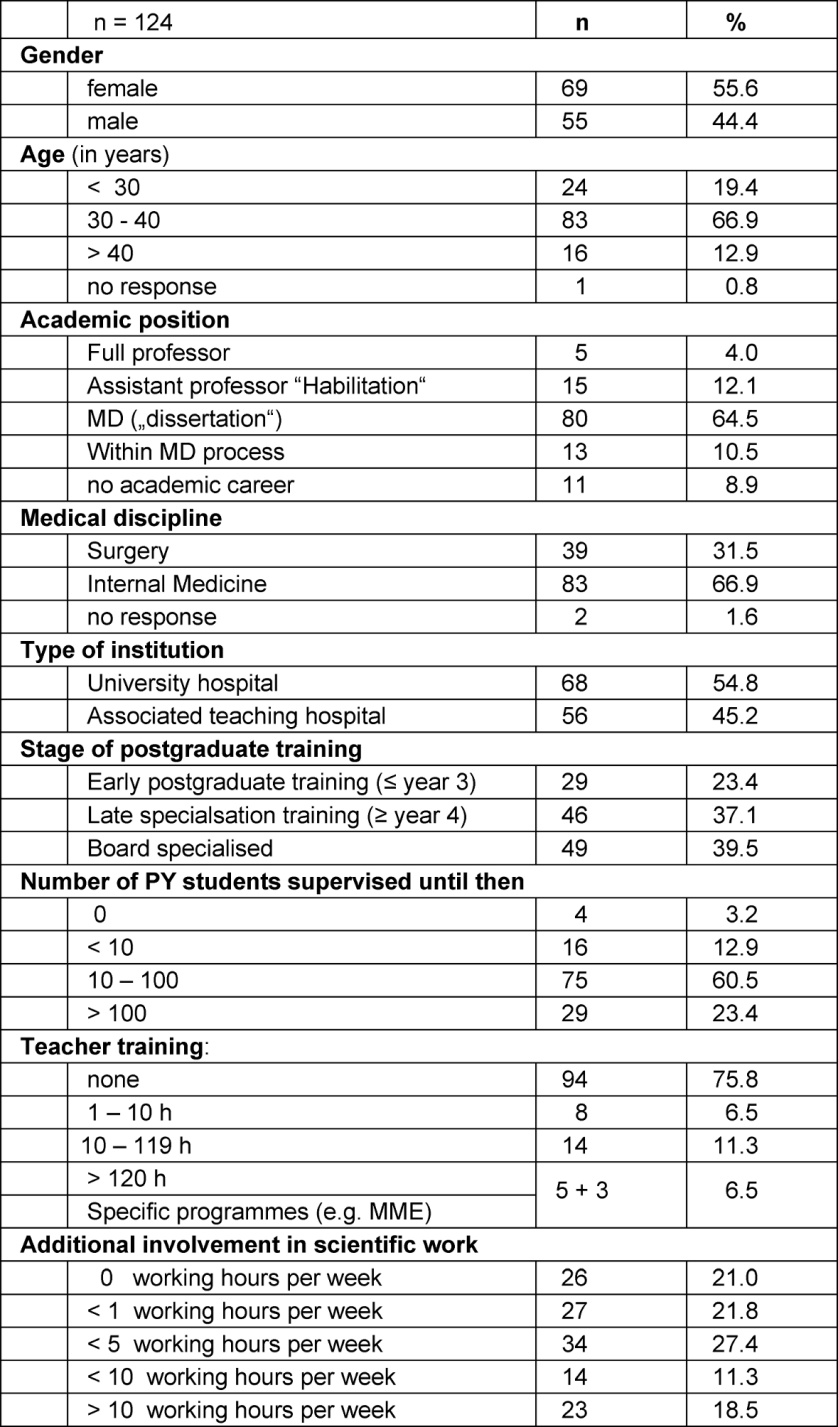
Characteristics of participating physicians
