# Supplementary material for: How do Supervising Clinicians of a University Hospital and Associated Teaching Hospitals Rate the Relevance of the Key Competencies within the CanMEDS Roles Framework in Respect to Teaching in Clinical Clerkships?
Source: GMS Z Med Ausbild. 2015 Aug 17;32(3):Doc33. doi: 10.3205/zma000975 (PMC4580442; doi:10.3205/zma000975)
Supplement: Supplemental material [file ZMA-32-33-s-001.pdf]

Questionnaire to rate physicians‘ roles and competencies according to their relevance as learning content during PY training

PART A:

Please select the option which is appropriate for your situation by clearly marking the respective letter on the (additional) answering sheet:

1) What is your gender?

A) male
B) female

2) Which category of age do you belong to?

A) < 30 years
B) 30- 40 years
C) > 40 years

3) Please indicate your (highest) academic degree.

A) professor
B) „habilitation“ (including final process)
C) dissertation (MD)
D) enrolled in MD programme
E) completed university education, no academic degree

4) In which medical discipline do you work at present ?

A) surgery
B) internal medicine

5) At which hospital are you employed at present?

A) Deutsches Herzzentrum München
B) Klinikum Bogenhausen oder Klinikum Schwabing
C) Klinikum rechts der Isar
D) Krankenhaus der Barmherzigen Brüder München
E) Rotkreuzklinikum München

6) What is your stage of medical training/education at present?

A) early postgraduate training (1st to 3rd year)
B) late postgraduate training (4th year, or higher)
C) board certification

7) During my physicians‘ occupation, I supervised (or co-supervised) the following number of PY students in total:

A) none
B) <10
C) 10-100
D) > 100

8) Did you ever attend a teacher training (faculty development programme)?

A) no

yes, in total

B) < 10 hours of teaching
C) 10-119 hours of teaching
D) ≥ 120 hours of teaching
E) A specific programme in medical education (e.g. postgraduate master)

9) How many working hours per week do you devote to scientific work in average (e.g. to manage clinical studies, basic research, educational research etc.) ?

A) 0 hours
B) ≤ 1 hours h
C) ≤ 5 hours
D) ≤ 10 hours
E) > 10 hours

PART B:

For the following questions we would like you to rate the relevance of certain competencies on a 5-point rating scale. Please, mark the respective letter on the (additional) answering sheet. The most extreme ratings code for:

„A“ = „fully agree“
„E“ = „fully disagree“

A) Role as „*Collaborator*“:

As Collaborators, physicians effectively work within a healthcare team to achieve optimal patient care.

Effectively work with other health professionals to prevent, negotiate, and resolve interprofessional conflict

10) How relevant is it to teach this competency to PY students ?
11) Do you actually teach this competency to PY students ?
12) How relevant is this competency for your personal daily work ?

Participate effectively and appropriately in an interprofessional healthcare team

13) How relevant is it to teach this competency to PY students ?
14) Do you actually teach this competency to PY students ?
15) How relevant is this competency for your personal daily work ?

B) Role as „*Health Advocate*“:

As Health Advocates, physicians responsibly use their expertise and influence to advance the health and well-being of individual patients, communities, and populations.

Physician responds to individual patient health needs and issues as part of patient care

16) How relevant is it to teach this competency to PY students ?
17) Do you actually teach this competency to PY students ?
18) How relevant is this competency for your personal daily work ?

Physician responds to the health needs of the communities that they serve

19) How relevant is it to teach this competency to PY students ?
20) Do you actually teach this competency to PY students ?
21) How relevant is this competency for your personal daily work ?

Physician identifies the determinants of health for the populations that they serve

22) How relevant is it to teach this competency to PY students ?
23) Do you actually teach this competency to PY students ?
24) How relevant is this competency for your personal daily work ?

Promote the health of individual patients, communities, and populations

25) How relevant is it to teach this competency to PY students ?
26) Do you actually teach this competency to PY students ?
27) How relevant is this competency for your personal daily work ?

C) Role as „*Medical Expert*“:

As Medical Experts, physicians integrate all of the CanMEDS Roles, applying medical knowledge, clinical skills, and professional attitudes in their provision of patientcentered care. Medical Expert is the central physician Role in the CanMEDS framework.

Use preventive and therapeutic interventions effectively

28) How relevant is it to teach this competency to PY students ?
29) Do you actually teach this competency to PY students ?
30) How relevant is this competency for your personal daily work ?

Function effectively as consultants, integrating all of the CanMEDS Roles to provide optimal, ethical and patient-centered medical care

31) How relevant is it to teach this competency to PY students ?
32) Do you actually teach this competency to PY students ?
33) How relevant is this competency for your personal daily work ?

Seek appropriate consultation from other health professionals, recognizing the limits of their expertise

34) How relevant is it to teach this competency to PY students ?
35) Do you actually teach this competency to PY students ?
36) How relevant is this competency for your personal daily work ?

Perform a complete and appropriate assessment of a patient

37) How relevant is it to teach this competency to PY students ?
38) Do you actually teach this competency to PY students ?
39) How relevant is this competency for your personal daily work ?

Establish and maintain clinical knowledge, skills and attitudes appropriate to their practice

40) How relevant is it to teach this competency to PY students ?
41) Do you actually teach this competency to PY students ?
42) How relevant is this competency for your personal daily work ?

Demonstrate proficient and appropriate use of procedural skills, both diagnostic and therapeutic.

43) How relevant is it to teach this competency to PY students ?
44) Do you actually teach this competency to PY students ?
45) How relevant is this competency for your personal daily work ?

D) Role as „*Scholar*“:

As Scholars, physicians demonstrate a lifelong commitment to reflective learning, as well as the creation, dissemination, application and translation of medical knowledge.

Maintain and enhance professional activities through ongoing learning

46) How relevant is it to teach this competency to PY students ?
47) Do you actually teach this competency to PY students ?
48) How relevant is this competency for your personal daily work ?

Critically evaluate medical information and its sources, and apply this appropriately to practice decisions

49) How relevant is it to teach this competency to PY students ?
50) Do you actually teach this competency to PY students ?
51) How relevant is this competency for your personal daily work ?

Contribute to the development, dissemination, and translation of new knowledge and practices

52) How relevant is it to teach this competency to PY students ?
53) Do you actually teach this competency to PY students ?
54) How relevant is this competency for your personal daily work ?

E) Role as „*Professional*“:

As Professionals, physicians are committed to the health and well-being of individuals and society through ethical practice, profession-led regulation, and high personal standards of behaviour.

Demonstrate a commitment to their patients, profession, and society through ethical practice

55) How relevant is it to teach this competency to PY students ?
56) Do you actually teach this competency to PY students ?
57) How relevant is this competency for your personal daily work ?

Demonstrate a commitment to their patients, profession and society through participation in profession-led regulation

58) How relevant is it to teach this competency to PY students ?
59) Do you actually teach this competency to PY students ?
60) How relevant is this competency for your personal daily work ?

Demonstrate a commitment to physician health and sustainable practice

61) How relevant is it to teach this competency to PY students ?
62) Do you actually teach this competency to PY students ?
63) How relevant is this competency for your personal daily work ?

F) Role as „*Manager*“:

As Managers, physicians are integral participants in healthcare organizations, organizing sustainable practices, making decisions about allocating resources, and contributing to the effectiveness of the healthcare system.

Participate in activities that contribute to the effectiveness of their healthcare organizations and systems

64) How relevant is it to teach this competency to PY students ?
65) Do you actually teach this competency to PY students ?
66) How relevant is this competency for your personal daily work ?

Manage their practice and career effectively

67) How relevant is it to teach this competency to PY students ?
68) Do you actually teach this competency to PY students ?
69) How relevant is this competency for your personal daily work ?

Allocate finite healthcare resources appropriately

70) How relevant is it to teach this competency to PY students ?
71) Do you actually teach this competency to PY students ?
72) How relevant is this competency for your personal daily work ?

Serve in administration and leadership roles, as appropriate

73) How relevant is it to teach this competency to PY students ?
74) Do you actually teach this competency to PY students ?
75) How relevant is this competency for your personal daily work ?

G) Role as „*Communicator*“):

As Communicators, physicians effectively facilitate the doctor-patient relationship and the dynamic exchanges that occur before, during, and after the medical encounter.

Develop rapport, trust, and ethical therapeutic relationships with patients and families

76) How relevant is it to teach this competency to PY students ?
77) Do you actually teach this competency to PY students ?
78) How relevant is this competency for your personal daily work ?

Accurately elicit and synthesize relevant information and perspectives of patients and families, colleagues, and other professionals

79) How relevant is it to teach this competency to PY students ?
80) Do you actually teach this competency to PY students ?
81) How relevant is this competency for your personal daily work ?

Accurately convey relevant information and explanations to patients and families, colleagues and other professionals

82) How relevant is it to teach this competency to PY students ?
83) Do you actually teach this competency to PY students ?
84) How relevant is this competency for your personal daily work ?

Develop a common understanding on issues, problems and plans with patients, families, and other professionals to develop a shared plan of care

85) How relevant is it to teach this competency to PY students ?
86) Do you actually teach this competency to PY students ?
87) How relevant is this competency for your personal daily work ?

onvey effective oral and written information about a medical encounter

88) How relevant is it to teach this competency to PY students ?
89) Do you actually teach this competency to PY students ?
90) How relevant is this competency for your personal daily work ?

Please, and finally, rank the above described roles according to their relevance for PY training.
The „medical expert“ role not ranked here, because of its integrative character.

(A= „highest priority“ B= „second priority” C= „third priority ” D „fourth priority ” E= „fifth priority ” no letter = lowest priority)

91. collaborator
92. health advocate
93. scholar
94. professional
95. manager
96. communicator
